# Structural and bioinformatics analysis of single-domain substrate-binding protein from *Rhodothermus marinus*

**DOI:** 10.1016/j.bbrep.2023.101611

**Published:** 2023-12-30

**Authors:** Ki Hyun Nam

**Affiliations:** College of General Education, Kookmin University, Seoul, 20707, Republic of Korea

**Keywords:** Substrate-binding protein, Single domain, Crystal structure, Flexibility, Metal binding, Modeling structure

## Abstract

Substrate-binding proteins (SBPs) are key elements in determining the substrate specificity and high affinity of the ATP-binding cassette uptake system. A typical SBP has two domains that recognize substrates and are responsible for the specific substrate delivery. Conversely, in GenBank, genes for SBPs constituting a single domain SBP are often found in vicinity of a methyl-accepting chemotaxis protein gene. However, the molecular function and mechanism of single domain SBPs are not fully elucidated. To understand their molecular functions, we performed a crystallographic study of single domain SBP from *Rhodothermus marinus* (RmSBP). RmSBP crystals were soaked in solution containing NaBr or HgCl_2_ and their structures determined at 1.75 and 2.3 Å resolution, respectively. RmSBP soaked in NaBr exhibited disorder of the α2-helix, β5-to β6-strand loop, and C-terminus region, showing the structural dynamic region of RmSBP. RmSBP soaked in HgCl_2_ showed that Hg^2+^ bound to Cys145 located between the α5-and α6-helices. The structural properties of RmSBP were compared with those of single domain SBP homologs. These results will contribute to continued identification of the molecular function and mechanism of single domain SBPs.

## Highlights

• Metal ion soaking experiments were conducted to elucidate the molecular function of RmSBP.
• Metal ion soaking induces flexibility in the helix of RmSBP.
• Mercury ions interacted with the Cys156 residue of RmSBP.
• An analysis of the amino acid sequence and structure of RmSBP-homologous proteins was performed.

## 1 Introduction

ATP-binding cassette (ABC) transporters are involved in the uptake and efflux of solutes across the cell membrane in both bacteria and eukaryotic cells [[1], [2], [3], [4]]. Substrate-binding proteins (SBPs) are key structural elements of the ABC transporter system that determine the substrate specificity and high affinity of the ABC uptake system [1,2]. Most prokaryotes possess SBP-dependent ABC transporters that recognize various ligands such as metal ions, amino acids, sugars, and peptides [1].

In nature, substrate-binding proteins (SBPs) exhibit a range of sizes, spanning from 25 to 70 kDa [5]. Although each SBP has low amino acid sequence similarity, the three-dimensional structural folds are conserved and have been divided into seven structural classes, according to their structural characteristics [5,6]. A typical SBP consists of two α/β domains, which are connected by a hinge region [5]. In general, when a substrate is not bound, an SBP maintains an open conformation, whereas when a substrate is bound to the substrate-binding pocket between the two α/β domains a closed conformation is formed between the two domains [[7], [8], [9]].

*Rhodothermus marinus* is a Gram-negative, marine, aerobic thermophilic eubacterium originally isolated from a submarine alkaline hot spring in Iceland [10]. It is moderately halophilic and grows optimally at 65 °C with 2% NaCl [10]. In GenBank, genes encoding SBPs with short lengths are found near the methyl-accepting chemotaxis protein gene [11]. Crystallographic analysis of *Rhodothermus marinus* SBPs (RmSBPs) showed that they had structural similarity to the C-terminal α/β domains of l-tryptophan-bound SBP from *Streptococcus pneumonia* (SpSBP) and phenylalanine-bound SBP from *Vibrio cholerae*) O1 biovar El Tor str. N16961 (VcSBP) but did not contain the N-terminal α/β domains [[11], [12], [13]]. The surface of the amino acid-binding position of SpSBP and VcSBP differed from the structurally identical position of RmSBP [11]. Computational analysis of the putative ligand binding sites of RmSBP indicated that these had identical structure positioning as the amino acid-binding site of SpSBP and VcSBP [12]. The putative substrate-binding site of RmSBP had a rigid structural folding while the β1–α2 and β5–β6 loops and extended C-terminal domains showed relatively high molecular flexibility [12]. On the other hand, the amino acid sequence of RmSBP shows a 20% similarity with that of ORF1ab polyprotein of severe acute respiratory syndrome coronavirus 2 (SARS-CoV-2) strain HKU-SZ-001_2020 [14]. Although previous crystallographic study of RmSBP shows the structural characteristics of a single-domain SBP, the functional role has yet to be elucidated.

To trace the molecular function of RmSBP, a ligand soaking experiment was conducted, and the crystal structures of RmSBP soaked in Br^-^ or Hg^2+^ solution were reported. Br-soaked RmSBP exhibited unexpected molecular flexibility in the α2-helix and C-terminus, with an observed disordered electron density map. Hg-soaked RmSBP revealed Hg^2+^ binding to a cysteine residue. Additionally, the amino acids and structure of RmSBP were compared with those of SBP homologs and their homologous structures. The structural flexibility observed in RmSBP through the soaking experiment and the structural comparison with single-domain SBP homologs will contribute to understanding the molecular function of single-domain SBPs.

## 2 Materials and methods

### 2.1 Sample preparation

RmSBP was cloned from *R. marinus* and purified as previously described [11]. Briefly, the gene encoding RmSBP (UniProt entry: D0MDR1, Pro22–Asn185) was cloned into the pET28a vector (Novagen). The corresponding vector containing the RmSBP gene was transformed into *Escherichia coli* BL21(DE3), which was cultured at 37 °C until an OD_600_ of 0.6–0.8. Protein expression was induced by adding 0.5 mM IPTG at 18 °C for 18 h. Cells were harvested by centrifugation and then disrupted via sonication. After removing the cell debris by centrifugation, the supernatant were loaded onto Ni-TNA resin in a column. The resin was washed with 50 mM Tris-HCl, pH 8.0, 200 mM NaCl, and 20 mM imidazole, and then the protein was eluted with 50 mM Tris-HCl, pH 8.0, 200 mM NaCl, and 300 mM imidazole. The expression tag, containing the N-terminal hexahistidine, was removed by incubation with thrombin at 4 °C for overnight. The protein was concentrated and loaded onto a Sephacryl S-100 column (GE HeathCare) equilibrated with 10 mM Tris-HCl, pH 8.0, and 200 mM NaCl. The eluted protein was concentrated for crystallization using a Centricon (Merck Millipore, cut-off: 10 kDa) concentrator.

### 2.2 Crystallization

Crystallization screens were performed using sitting-drop vapor diffusion method at 20 °C. A purified RmSBP solution (∼20 mg/ml, 1 μL) was mixed with a crystallization solution (1 μL) from a commercially available crystal screen kit. Microcrystals of RmSBP were obtained in the following crystallization conditions: (i) Index D8 [0.1 M HEPES pH 7.5, 25% (w/v) polyethylene glycol 3,350] and (ii) Index G11 [0.1 M Bis-Tris, pH 6.5, 0.2 M magnesium chloride and 25% (w/v) polyethylene glycol 3,350] from a Hampton Research crystallization kit. Suitable crystals for X-ray diffraction experiment were obtained with hanging-drop vapor diffusion method at 20 °C by scale up of the above crystallization solutions.

### 2.3 Data collection

X-ray diffraction data were collected on the 11C beamline at Pohang Accelerator Laboratory (Pohang, Republic of Korea) [15]. For metal soaking, the RmSBP crystals obtained in Index D8 and G11 conditions were immersed in a reservoir solution containing 0.1 mM NaBr or HgCl_2_ for 30 s, respectively, and then cryoprotected using a cryoprotectant solution containing the reservoir solution supplemented with 20% (v/v) glycerol for 5 s. Diffraction data were recorded on Pilatus 6 M and were processed using the HKL2000 program [16].

### 2.4 Structure determination

The phasing problem was solved using the molecular replacement method implemented in MOLREP [17], a program embedded in the CCP4i suite [18]. The crystal structure of RmSBP at pH 8.0 (PDB code: 5Z6V) [11] used as the search model. Manual model building was performed in Coot (version 0.9.6) [19] based on visual inspection of the electron density maps. The 2Fo-Fc omit map and anomalous difference map analyses were performed to confirm the presence of the mercury atom in RmSBP-Hg. Structure refinements were carried out with phenix.refine within the PHENIX (version 1.20.1_4487) [20]. Water molecules were automatically added during the refinement process using the default parameters within PHENIX [20]. The geometry of the final model structure was validated using MolProbity [21]. The atomic coordinates and structure factors for the final model have been deposited in the Protein Data Bank under accession codes 8WGK (NaBr soaking) and 8WGL (HgCl_2_ soaking).

### 2.5 Bioinformatics

RmSBP homologs were identified via BLAST searches on UniProt (https://www.uniprot.org/blast). UniProtKB reference proteomes and Swiss-Prot were utilized as the target databases for BLAST searches. The blast searching parameters included blastp, an E-threshold of 10, and the BLOSUM62 matrix. Sequence alignment was conducted using Clustal Omega [22]. The structure-based sequence alignment was visualized with ESPript [23]. Information regarding the amino acids in the signal peptide of RpSBP (UniProt entry: A0A1M6PUA2), RbSBP (A0A1V0DID0), and SrSBP (Q2RZD5) was obtained from the UniProt site (http://uniprot.org). Homology models for RpSBP, RbSBP, and SrSBP were retrieved from the AlphaFold [24] protein structure database (https://alphafold.ebi.ac.uk/).

## 3 Result and discussions

Most prokaryotes possess SBP-dependent ABC transporters, where the SBPs recognize a variety of ligands such as metal ions, amino acids, sugars, and peptides [1]. To investigate whether RmSBP belongs to the group of SBPs that recognize metals or amino acids as ligands, a soaking experiment was performed using metal ions and amino acids. To prevent nonspecific ligand binding, RmSBP crystals were soaked in the reservoir solution supplemented with a final low concentration of 0.1 mM metal ions (K^+^, Ca^2+^, Mn^2+^, Fe^2+^, Co^2+^, Ni^2+^, Cu^2+^, Zn^2+^, Hg^2+^, or Br^-^) or an amino acid mixture (2.5 mM Ala, Arg, Asp, Glu, Gly, His, Ile, Leu, Lys, Met, Phe, Pro, Ser, Thr, Tyr, Val, and 1.25 mM Cys) for 30 s. Subsequently, diffraction data were collected after cryoprotection. The crystal structures of RmSBP soaked in NaBr and HgCl_2_ had different structural characteristics from the previously reported RmSBP (see below), but no significant structural changes were found in crystals soaked in other metal ions and amino acids.

The crystal of RmSBP soaked with NaBr (RmSBP-Br) belongs to the orthorhombic P2_1_2_1_2_1_ space group with unit cell parameters a = 92.84, b = 97.86, and c = 101.98 Å, containing one RmSBP molecule per asymmetric unit. This space group and unit cell parameter is similar with previous report RmSBP determined at pH 5.5 (PDB code: 6K1W) and pH 7.5 (6K1Y) [12]. However, the crystal structure of RmSBP-Br showed three distinct disordered regions, indicating a potential structurally flexible region (see below). The crystal of RmSBP soaked with HgCl_2_ (RmSBP-Hg) belongs to the monoclinic P2_1_ space group with the unit cell parameters a = 92.84, b = 97.86, c = 101.98 Å, and β = 90°, containing two RmSBP molecules in the asymmetric unit, which is a new crystal form and diffracted up to 2.0 Å. The two RmSBP-Hg molecules in the asymmetric unit clearly showed an electron density map for building all amino acids from Glu27 to Leu182. The crystal structures of RmSBP-Br and RmSBP-Hg were determined at 1.75 and 2.30 Å resolution, respectively (Table 1). The R_work_/R_free_ values of final model structure of RmSBP-NaBr and RmSBP-HgCl were 22.23/26.08 and 19.73/25.00, respectively.

**Table 1:** Data collection and refinement statistics.

Data collection	NaBr soaking	HgCl_2_ soaking
X-ray source	Beamline 7A, PLS-II	Beamline 7A, PLS-II
X-ray energy (eV)	12659	12659
Space group	P2_1_2_1_2_1_	P2_1_
Cell dimension		
a, b, c (Å)	46.231, 49.129, 57.518	49.645, 45.209, 57.041
α, β, γ (°)	90.00,	90.00, 91.78, 90.00
Resolution (Å)	50.0–1.75 (1.78–1.75)	50.0–2.3 (2.34–2.30)
Unique reflections	13670 (681)	11062 (570)
Completeness (%)	97.4 (100.0)	96.4 (100.0)
Multiplicity	3.8 (3.9)	2.7 (2.8)
I/sigma	36.47 (5.63)	21.83 (5.62)
R_merge_	0.064 (0.355)	0.080 (0.373)
**Refinement**		
Resolution (Å)	21.87–1.75	24.11–2.30
R_work_^a^	0.2223	0.1973
R_free_^b^	0.2608	0.2500
RMS deviations		
Bonds (Å)	0.006	0.003
Angles (°)	0.838	0.663
*B* factors (Å^2^)		
Protein	29.48	35.49
Water	35.57	37.91
Metal ion		40.01
Ramachandran plot		
Favored (%)	97.73	96.75
Allowed (%)	2.27	2.92
Disallowed (%)	0.00	0.32

^1^ Values for the outer shell are given in parentheses.

a R_work_ = Σ||Fobs| Σ |Fcalc||/Σ|Fobs|, where Fobs and Fcalc are the observed and calculated structure factor amplitudes, respectively.

b R_free_ was calculated as R_work_ using a randomly selected subset (5%) of unique reflections not used for structural refinement.

**Fig. 1:**
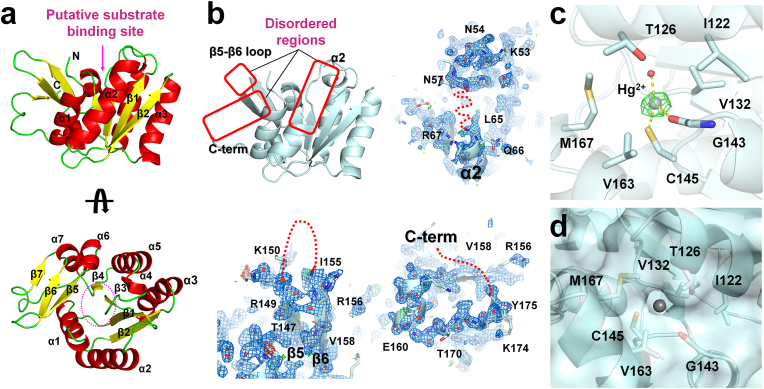
Analysis of crystal structure of SBP from *R. marinus*. (a) Cartoon representation of the single-domain RmSBP. The putative substrate binding site is indicated by a pink dot circle. (b) 2Fo-Fc (blue mesh, 1σ) and Fo-Fc (green mesh, 3 σ; red mesh, −3 σ) electron density maps for disordered region for the α2-helix (Arg58–Gln64), loop between β5-and β6-strands (Asp151–Gly154), and C-terminus (Gln176–Leu182) from RmSBP-Br. The disordered regions in RmSBP-Br were indicated by red boxes or red dot lines. (c) 2Fo–Fc omit electron density map (green mesh, 6 σ) for Hg^2+^ binding on Cys145 from RmSBP-Hg. (d) Surface structure of the Hg^2+^ binding site of RmSBP-Hg-B. The Hg^2+^ was not buried by the amino acids of RmSBP. (For interpretation of the references to colour in this figure legend, the reader is referred to the Web version of this article.)

The overall structure of RmSBP showed the single α/β fold (Fig. 1A), which is identical with that previous reported, although the molecular flexibility was distinct. The crystal structure of RmSBP-Br showed the three disordered regions at the α2-helix (Arg58–Gln64), the loop between β5-and β6-strands (Asp151–Gly154), and the C-terminus (Gln176–Leu182) (Fig. 1B). These three disordered regions were shown to have relative flexibility in the previous crystal structure analysis [12], but were clearly disordered in RmSBP-Br. These three disordered regions in RmSBP-Br are exposed to solvent in the P2_1_2_1_2_1_ crystal packing. Although NaBr was used for soaking, no electron density map corresponding to a Br ion was observed, and it is possible that NaBr had diffused in the solvent regions in crystal packing, increasing the flexibility of the three disordered regions. This indicates that these regions of RmSBP are likely to be dynamic in a tentatively solution state. However, the position of three disordered regions of RmSBP was not directly related to the previously predicted substrate-binding site of RmSBP [12]. Therefore, these regions may be involved in interactions with potential partner molecules or receptor proteins.

The crystal structure of RmSBP-Hg showed a strong positive Fo–Fc electron density (>7 sigma) on the Cys145 in the loop between the α5-helix and β5-stand, which surrounds the α5-and α6-helices (Fig. 1C). Hg^2+^ interacted with Cys145 and a water molecule at an average distance of 2.57 and 2.27 Å, respectively. Furthermore, Hg^2+^ interacted with the main chain of the O atom of Gly132 and N atom of Val132 at average distance of 3.42 and 3.48 Å, respectively. Hg^2+^ was surrounded by Ile122, Thr126, Val132, Val163, and Met167 residues. In the RmSBP-Hg-A molecule, Hg^2+^ was buried by neighboring amino acids, but in the surface structure of the RmSBP-Hg-B molecule, the conformations of the amino acids around Hg^2+^ were slightly different, and Hg^2+^ was observed through the hole in the surface structure (Fig. 1D). The Cys145 of RmSBP, where Hg^2+^ bound, was located on the side of the α/β domain, which is different from the previously expected substrate-binding site. Mercury readily reacts with sulfur-containing compounds such as cysteine [25,26]. Mercury is often used to solve phasing problems using heavy atom binding to cysteine residues in protein crystals [27]. Accordingly, further studies will be needed to see if the heavy metals bound to Cys145 in RmSBP are biologically meaningful.

**Fig. 2:**
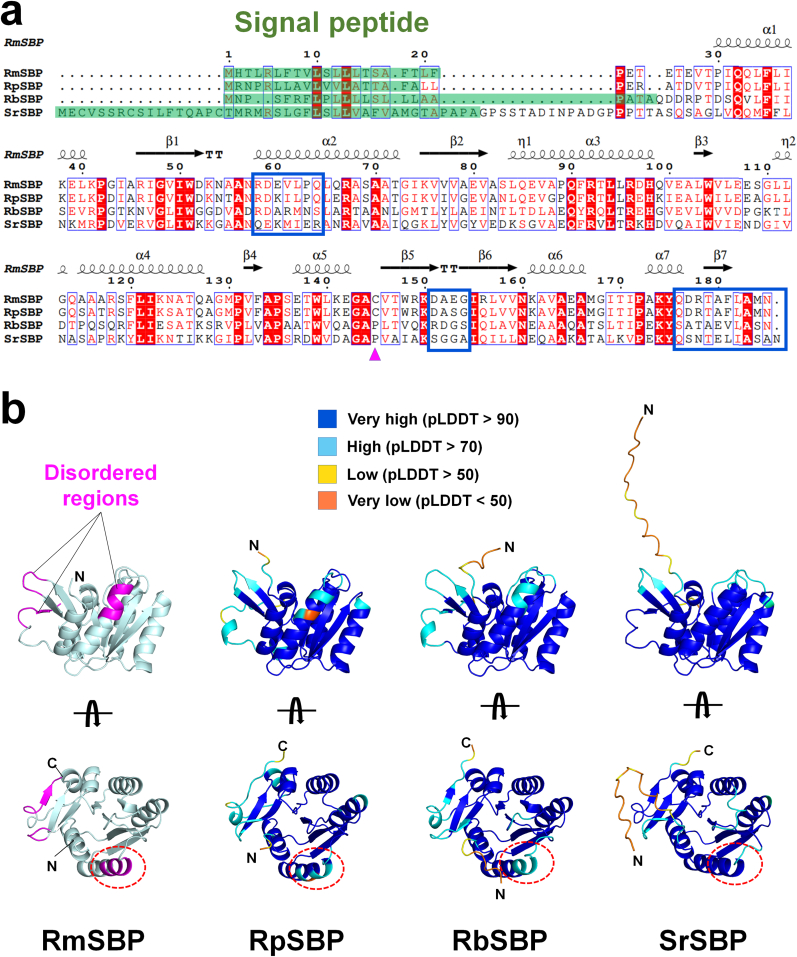
Comparison of amino acids and structure of RmSBP with homologous proteins. (a) Structure-based sequence alignment of RmSBP (UniProt: D0MDR1) with putative ABC transport system SBP from *Rhodothermus profundi* (RpSBP, A0A1M6PUA2), uncharacterized protein from *Rhodothermaceae bacterium* RA (RbSBP, A0A1V0DID0), and uncharacterized protein from *Salinibacter ruber* strain DSM 13855 /M31 (SrSBP, Q2RZD5). Conserved residues are in red boxes, and the homologous residues are in unfilled boxes with red letters. The mercury-binding residue in RmSBP is indicated by a pink triangle. (b) Structure comparison of RmSBP with modeling structures of RpSBP, RbSBP, and SruSBP generated by AlphaFold2. The flexible α2-helix region in RmSBP induced by Br-soaking was indicated by a red dot circle. (For interpretation of the references to colour in this figure legend, the reader is referred to the Web version of this article.)

To understand whether these structural features of RmSBP are conserved in homologous proteins, the amino acids and structure of RmSBP were compared with those of putative ABC transport system SBP from *Rhodothermus profundi* (RpSBP, Accession No.: A0A1M6PUA2), uncharacterized protein from *Rhodothermaceae bacterium* RA (RbSBP, A0A1V0DID0), and uncharacterized protein from *Salinibacter ruber* strain DSM 13855 /M31 (SrSBP, Q2RZD5) (Fig. 2A). The number of amino acids in RmSBP (185) is the same as that in RpSBP (185) and RbSBP (185), whereas the number in RmSBP is less than that in SrSBP (224) (Fig. 2A). The predicted signal peptides of RmSBP, RpSBP and RbSBP contain 21, 19, and 23 amino acids, respectively, whereas that of SrSBP contains 43 amino acids. Accordingly, RsSBP, RpSBP, RbSBP, and SrSBP contained 164, 166, 162, and 201 amino acids, respectively, excluding the signal peptides. RmSBP, excluding signal peptides, shows sequence identities of 86.6% (164 residue overlapping), 39.5% (157 residues), and 38.6% (153 residues), respectively, with RpSBP, RbSBP, and SrSBP. The Cys145 with Hg^2+^ binding in RmSBP is conserved in RpSBP but has been replaced by proline in the other SBPs (Fig. 2A). This result indicates that Hg^2+^ may not be directly involved in the biological function of SBP. Mercury readily reacts with sulfur-containing compounds such as cysteine and often exists as a thiol S-conjugate [28]. Accordingly, the mercury associated with RmSBP-Hg is most likely a result of the affinity between Hg^2+^ and cysteine due to atomic properties, rather than biological function. The sequences of the disordered α2-helix (Arg58–Gln64), loop between the β5-and β6-strands (Asp151–Gly154), and C-terminus (Gln176–Leu182) in RmSBP-Br are similar to those of RpSBP and potentially have a similar molecular flexibility. Although some amino acid similarities were seen in other SbSBP, these were not identical, and they were considered to exhibit different molecular flexibility.

The model structures of RpSBP, RbSBP, and SrSBP exhibit high structural similarity to RmSBP at the α/β-fold (Fig. 2B). SrSBP contains more amino acids than the other SBPs, and the corresponding region corresponds to a flexible loop that is uninvolved in folding at the N-terminal. The homology structures generated by AlphaFold produce a per-residue model confidence score (pLDDT) between 0 and 100 [24]. A pLDDT score of >90 indicates high accuracy, 70–80 suggests general accuracy with some uncertainty, and 50–70 signifies uncertainty and caution [24]. In the model structures of RpSBP and RbSBP, equivalent positions of the disordered regions of RmSBP showed the pLDDT values of <90%. In the model structure of SrSBP, the loop region between β5-and β6-strands of SrSBP shows a relatively low pLDDT value, whereas the other region in SrSBP shows a pLDDT value of >90. Accordingly, RmSBP exhibits a structural α/β-fold structural similarity to other single-domain SBPs, and in particular, the flexible region of RmSBP shows similarity to that in RpSBP and RbSBP.

While metal ion and amino acid soaking experiments were performed on RmSBP, unexpected structural mobility of RmSBP and binding of Hg^2+^ to Cys153 were discovered, providing useful information for understanding the structural properties of RmSBP. However, a biologically meaningful substrate for RmSBP, the primary purpose of the soaking experiment, was not identified in this study. Given that the experiment covered only a limited number of metal ions and amino acids, affinity for other types of metals and amino acids should be measured in future studies through additional soaking experiments or biochemical assays. Furthermore, potential substrate candidates, such as sugars or peptides, may need to be screened in future experiments. Additionally, considering that single-domain SBP contains only one domain, extensive studies will be necessary to determine whether other partner molecules are required.

## 4 Conclusions

The molecular function of the unique feature of single-domain SBP, consisting of a single α/β domain, has remained unknown. To investigate the molecular function of this single-domain SBP, a ligand-soaking experiment was conducted for RmSBP. In RmSBP-Br, unprecedented disordered regions of RmSBP were observed, revealing structural properties that contribute to molecular flexibility. In RmSBP-Hg, binding of Hg^2+^ to Cys145 was observed, necessitating further confirmation to establish its biological significance. Amino acid and structural comparisons of RmSBP indicated similarity with homologous single-domain SBPs. These structural insights will serve as valuable information for comprehending the functionality of single-domain SBPs in future studies.

## CRediT authorship contribution statement

**Ki Hyun Nam:** Conceptualization, Data curation, Funding acquisition, Methodology, Resources, Writing – original draft.

## Declaration of competing interest

The authors declare that they have no known competing financial interests or personal relationships that could have appeared to influence the work reported in this paper.

## Data Availability

Data will be made available on request.
